# Nanocircularity in Agriculture with BioC-Dots: Vinasse-Derived Carbon-Dots and Their Usage as Potential Metal Ion Sensors

**DOI:** 10.1021/acsomega.4c11597

**Published:** 2025-08-16

**Authors:** Ulisses N. de Morais, Eralci M. Therézio, Ailton J. Terezo, Adriano B. Siqueira

**Affiliations:** † Institute of Chemistry, 67826 Rede MT-NanoAgro/GENMAT-Federal University of Mato Grosso (UFMT), Cuiabá, Mato Grosso 78060-900, Brazil; ‡ Institute of Physics, Federal University of Mato Grosso, Cuiabá, Mato Grosso 78060-900, Brazil; § National Institute of Science and Technology in Nanotechnology for Sustainable Agriculture, INCTNanoAgro, Cuiaba 78060-900, Brazil

## Abstract

The sugar cane refining industry is crucial in producing sugar and biofuel ethanol. However, as demand for these products has grown, so has the generation of waste, with vinasse emerging as the primary pollutant. This study investigates the potential of utilizing vinasse as a raw material to produce carbon dots (BioC-dots), a high-value product. Application of the synthesized BioC-dots in metal ion detection is yet to be explored. Luminescent nanocarbons (BioC-dots) were successfully synthesized from vinasse using a green synthesis method and characterized through transmission electron microscopy (TEM) imaging, UV–vis spectroscopy, and photoluminescence spectroscopy, which revealed a quantum yield ranging from 2.5% to 19%. TG-DTA curves and FTIR spectra revealed a remarkable thermal behavior and main functional groups of the surface of nanocarbon. The interaction of metals with BioC-dots in solution demonstrated their potential as effective sensors for cobalt and zinc ions in aqueous environments, showing detection limits of 13.57 and 18.22 mmol L^–1^, respectively.

## Graphical abstract

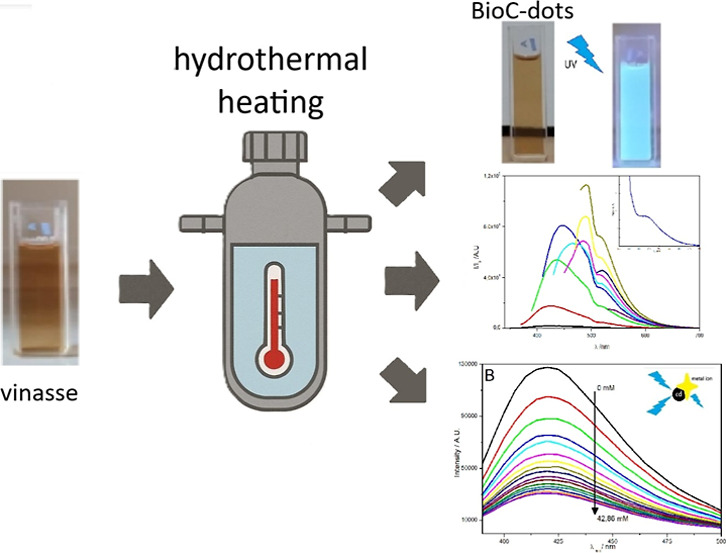

## 1 Introduction

The agro–industrial complex in Brazil is a significant part of its economy and a primary focus of studies, including research on the use and combination of micronutrients to achieve higher yields throughout the year.^1^ The sugar cane industry is one of the most critical sectors of the agro-industrial complex, responsible for producing essential products such as ethanol and sugar. Brazil is considered a major producer of ethanol in the global market, having produced approximately 26,69 billion liters of sugar cane alcohol during the 2023/24 harvest, as reported by the National Supply Company of Brazil (CONAB).^2^

The sugar cane processing process yields a diverse range of byproducts. Some residues, such as filter cakes and bagasse, from the refining and processing chain have already been reported to have uses as energy sources, animal rations, and fertilizers. This adds even more value to the sector, creating a circular economy and lowering production costs. At the end of the distillation process, a waste effluent known as vinasse is produced, which serves as a target for studies on possible uses or treatments.^3^ Produced during the distillation of the sugar cane juice, at around 10–16 L for every liter of ethanol produced, vinasse is considered one of the most polluting residues of the sugar cane processing chain, with its main organic components being glycerol, lactic acid, ethanol, and acetic acid, along with the presence of phenols and melanoidins, low pH value, and high chemical and biochemical oxygen demand (COD and BOD) values.^4^ The high amount of wastewater produced and its polluting nature complicate its potential usage cases due to the need to treat wastewater for specific applications. Multiple wastewater treatment methods have been explored to reduce the polluting factor with a focus on lowering the content of phenolic compounds, such as tannic and humic acids, found in the effluent. Anaerobic treatment methods, including fixed-bed, fluidized, and upflow anaerobic sludge blanket (UASB) reactors, have yielded satisfactory results, as have aerobic treatment methods utilizing fungi, such as *Pleurotus ostreatus* *.* ^5^

The conversion of biomass into new materials presents an innovative approach that lowers preparation costs and effectively addresses waste disposal challenges. It is very sustainable. Its inherent flexibility allows for various applications, particularly in doping or functionalization processes. Nanocarbon luminescent (carbon dots, CDs) sourced from biomass offer numerous advantages, but they are economically viable, biocompatible, and exhibit low toxicity, ensuring that they pose minimal environmental risk. This combination of attributes paves the way for the widespread application of biomass-derived CDs across various industries, making them a promising alternative to sustainable materials. By harnessing the potential of biomass, we can contribute to an eco-friendly future while simultaneously benefiting from effective resource management.^6^

Carbon dots are nanoscale particles derived from carbon-rich raw materials such as biomass. Due to their unique properties, carbon dots are the target of various application studies. With a usual size between 2 and 10 nm, carbon dots are the focus of multiple studies of possible applications, especially in sensory areas and in agriculture.^7^ Among the more interesting aspects of carbon dots, their fluorescence is particularly relevant, with the wavelength of the emission configuration being a crucial aspect for a desired application. The fluorescence mechanism of carbon dots remains a focus of significant studies, with its exact origin still being debated in the literature. Two major theories help clarify the phenomenon: surface state theory and the quantum confinement effect.^8^

The luminescent characteristics of carbon dots make their use in detection and monitoring applications attractive, with various studies published that utilize carbon dots synthesized from different biomass sources for detecting metals, anions, and diverse molecules with a satisfactory level of detection (LOD). Jayaweera et al. synthesized carbon dots using durian shell waste as a raw material, employing the traditional hydrothermal synthesis method for detecting Fe­(III) ions. The resulting BioC-dots exhibited a luminescence extinction system with a limit of detection (LOD) of 128 nmol L^–1^.^9^ Yu et al. utilized carbon dots synthesized from lettuce leaves to create an on–off system for detecting methimazole, utilizing silver ions to quench the luminescence of carbon dots’ luminescence. Introducing methimazole into the system would cause the return of photoluminescence due to the interaction of the medication with silver ions located at the surface of the carbon dots, resulting in a limit of detection (LOD) of 8 nmol/L.^10^ Valarmathy and Sudhaparimala synthesized carbon dots, using *Moringa oleifera* leaves as precursors, for use as a heavy-metal ion sensor. The produced BioC-dots exhibited a LOD of 1.28 mmol L^–1^.^11^

Therefore, vinasse has excellent potential for synthesizing carbon dots (BioC-dots or BCD-V). C-dots have already been prepared from vinasse (CQD-V), showing antioxidant and antimicrobial activity and being considered a noncytotoxic nanomaterial. This work demonstrated the feasibility of using vinasse as a raw material for the one-pot synthesis of carbon dots (BioC-dots or BCD-V) via a hydrothermal route without dilution or presynthesis treatment and its potential application as a metal ion detector.

## 2 Materials and Methodology

### 2.1 Synthesis

Two Teflon-lined stainless-steel autoclaves were loaded with 40 mL of vinasse and then heated at 210 °C for 6, 12, and 24 h. 1 mg of l-cysteine was added to an independent vinasse sample and heated under the same conditions for 24 h to produce functionalized BioC-dots and to evaluate the possible improvements of the produced BioC-dots. The obtained products were filtrated in a Millipore system using 0.45 and 0.22 μm membranes and submitted to ultrasonic treatment. The produced BioC-dots were designated as BCD-V 1, 2, 3, and 4 and then refrigerated at 5 °C for further characterization.

### 2.2 Characterization

The pH and conductivity measurements of the various solutions were meticulously recorded utilizing an AKSO Combo 5 multiparameter meter, ensuring an accurate assessment of these vital parameters.

The absorption spectra within the ultraviolet and visible regions (UV–vis) were generated by employing a PerkinElmer model Lambda 265 spectrophotometer. This spectrophotometer spans a comprehensive wavelength range of 190–1100 nm, allowing for detailed analysis of the samples’ light-absorbing properties.

For the emission spectra of the BioC-dots, a sophisticated ISS PC1 Photon Jobin Yvon spectrofluorometer powered by a robust 500 W Xe lamp was used. To achieve precise measurements, 0.5 mm slits were employed in both the emission and the excitation monochromators. The samples were carefully placed into quartz cuvettes featuring an optical pathway of just 1 mm to enable an optimal light interaction.

To investigate the thermal decomposition characteristics of the untreated vinasse and the synthesized BioC-dots, thermogravimetric differential thermal analysis (TG-DTA) was performed. This analysis was conducted on a Shimadzu DTG-60H thermal analyzer under a controlled environment of compressed air, with a flow rate of 100 mL min^–1^. During this critical evaluation, the temperature was ramped at 10 °C min^–1^ using alumina crucibles to contain the samples.

The infrared absorption spectrum was acquired through a PerkinElmer spectrophotometer, model Spectrum 100, which features a resolution of 4 cm^–1^. This analysis was conducted across the 4000–600 cm^–1^ spectral range, utilizing the attenuated total reflectance (ATR) technique to enhance the sensitivity and accuracy in identifying molecular vibrations within the samples.

The transmission electron microscopy (TEM) technique was employed to observe and specify the formation of clusters and the size of the synthesized BioC-dots. SEM analysis was performed on a Quanta 200 FEG FEI microscope, model 14 2006. The sample was metalized with a thin layer of gold using a Shimadzu ICSO metallizer, where a small amount of each sample was placed on an aluminum sample holder containing a carbon adhesive.

### 2.3 Metal Biosensor

BioC-dots were evaluated to sense Mn­(II), Co­(II), and Ni­(II) metal ions. To evaluate the detection efficiency of metal ions, three stock aqueous solutions were prepared at 0.1 mol L^–1^ by diluting cobalt­(II)­nitrate hexahydrate, manganese­(II)­chloride tetrahydrate, and nickel­(II)­sulfate hexahydrate in water and subsequently diluting each solution separately using the produced BCD-V 4 solution.

The results were based on the quenching effect caused by the interaction between the BioC-dots and the metal species in the solution. The PL spectra were recorded as exciting BioC-dots functionalized at λ = 360 nm. The quenching was evaluated using the Stern–Volmer relationship, described in eq 1.^12^

1 $$\frac{M^{0}}{M}=1+K_{SV}\left[Q\right]$$

*M* ^0^ denotes the emission intensity in the absence of the metal ion, while *M* represents the emission intensity in its presence. The variable [Q] indicates the concentration of the metal ion, and *K*_SV_ refers to the Stern–Volmer constant.^13^

## 3 Results and Discussion

### 3.1 BioC-Dots Synthesis

The type of synthesis influenced the formation of luminescent nanocarbons, which could be seen under black light emission accompanied by blue light (Figure 1).

**Figure 1:**
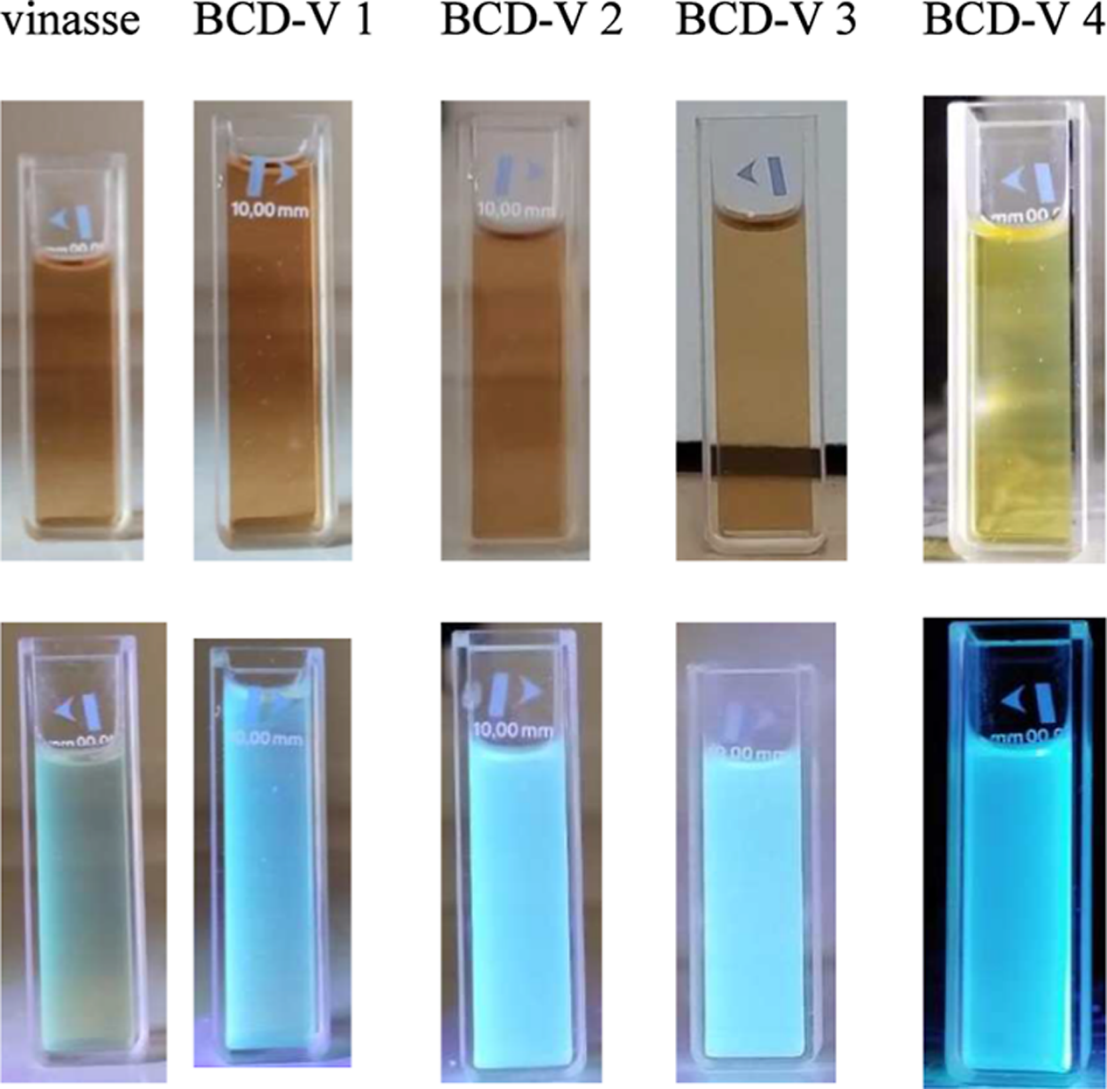
Photographic images of vinasse and BioC-dots under sunlight (above) and UV-light (below) (λ = 395 nm).

The pH values of the BioC-dot solutions, alongside those of the vinasse, are compiled in Table 1. Notably, the pH measurements recorded for the vinasse align well with the values documented from earlier harvests, demonstrating consistent findings.^14^ The BioC-dot solution was tested, and the pH was lowered and increased. Photoluminescence extinction was observed in all of the tests, indicating that the solution is in the ideal pH for better emission intensity. The acidic characteristic of the samples is explained by the predistillation fermentation process, in which yeast is maintained at an acidic pH to avoid the growth of invasive bacteria, along with the presence of acidic byproducts of ethanol production. Sugar cane vinasse samples have high glycerol, lactic acid, ethanol, acetic acid content and oxalate, acetate, melanoidin, and phenolic compounds.^15^

**Table 1:** Absorbance and pH Values of the Vinasse and BioC-Dots Samples

sample	λ_max_/nm	absorbance	pH	half-height length
vinasse	275	1.34	4.74	77.26
BCD-V 1	262	1.57	4.77	113.6
BCD-V 2	270	1.06	4.91	87.07
BCD-V 3	263	0.859	4.78	94.89
BCD-V 4	257	1.49	4.07	68.83

### 3.2 UV–Vis Spectra

The UV–vis spectra of vinasse (Figure 2) report high absorbance values at 275 nm, referring to π → π* transitions of C = C groups present in the raw material. The vinasse sample presents a yellowed coloration under daylight and a bluish coloration under UV light (λ = 395 nm), indicating the presence of fluorescent compounds. A possible explanation of the luminescence in the untreated sample of vinasse is the possible BioC-dot formation in the distillation process and during the storage in a dark vase at room temperature, with a noticeable build-up in internal pressure in the storage vase.

**Figure 2:**
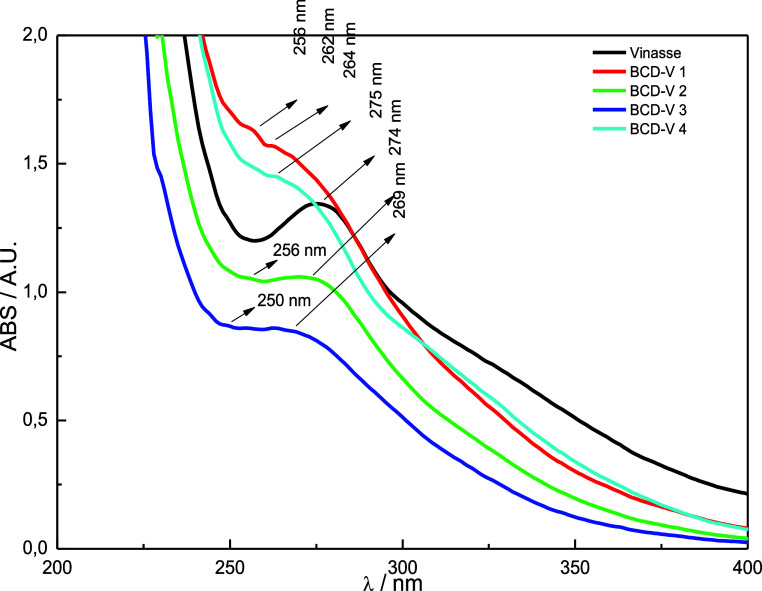
UV–vis spectra of vinasse and BioC-dots samples.

The UV–vis absorption spectra of the synthesized BioC-dots in the traditional hydrothermal reactor (Figure 2) presented absorption bands in the 250–270 nm range, corresponding to π → π* of C = C and C = N bonds in the samples. Under daylight, the solutions are orange and have a more intense bluish coloration when under black light (λ = 395 nm). The BCD-V 4 sample presented higher absorption values when diluted to the same proportions as those of the other samples. However, the absorption spectra remained relatively like the one presented by the 6 h synthesis. The absorption of the materials is organized in Table 1.

### 3.3 Emission Spectra

The emission studies conducted on the vinasse samples revealed a notably high emission intensity when they were excited with light at a wavelength of 460 nm (Figure 3). This excitation resulted in a prominent emission peak observed at 530 nm, predominantly corresponding to green light. In comparison, the BioC-dot samples exhibited either an enhanced emission intensity or shifts in their emission peaks, as illustrated in Figure 4. Photoluminescence analyses of the BCD-Vs were conducted over one year, with no change in quantum yield observed, indicating the photostability of the material during this time.

**Figure 3:**
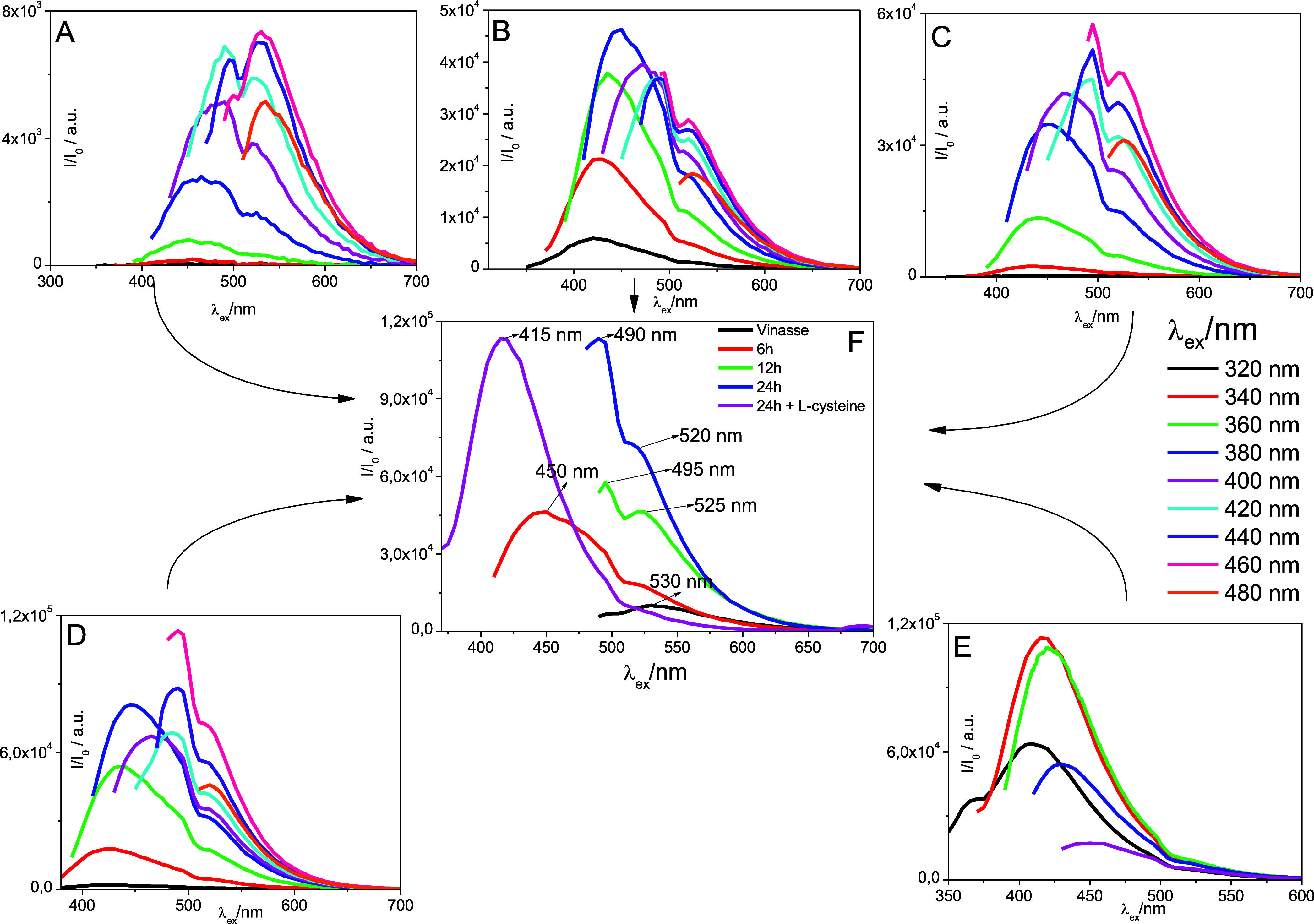
Emission spectra of vinasse (A), BCD-V 1 (B), BCD-V 2 (C), BCD-V 3 (D), BCD-V 4 (E) and the maximum emission intensity of each sample (F).

**Figure 4:**
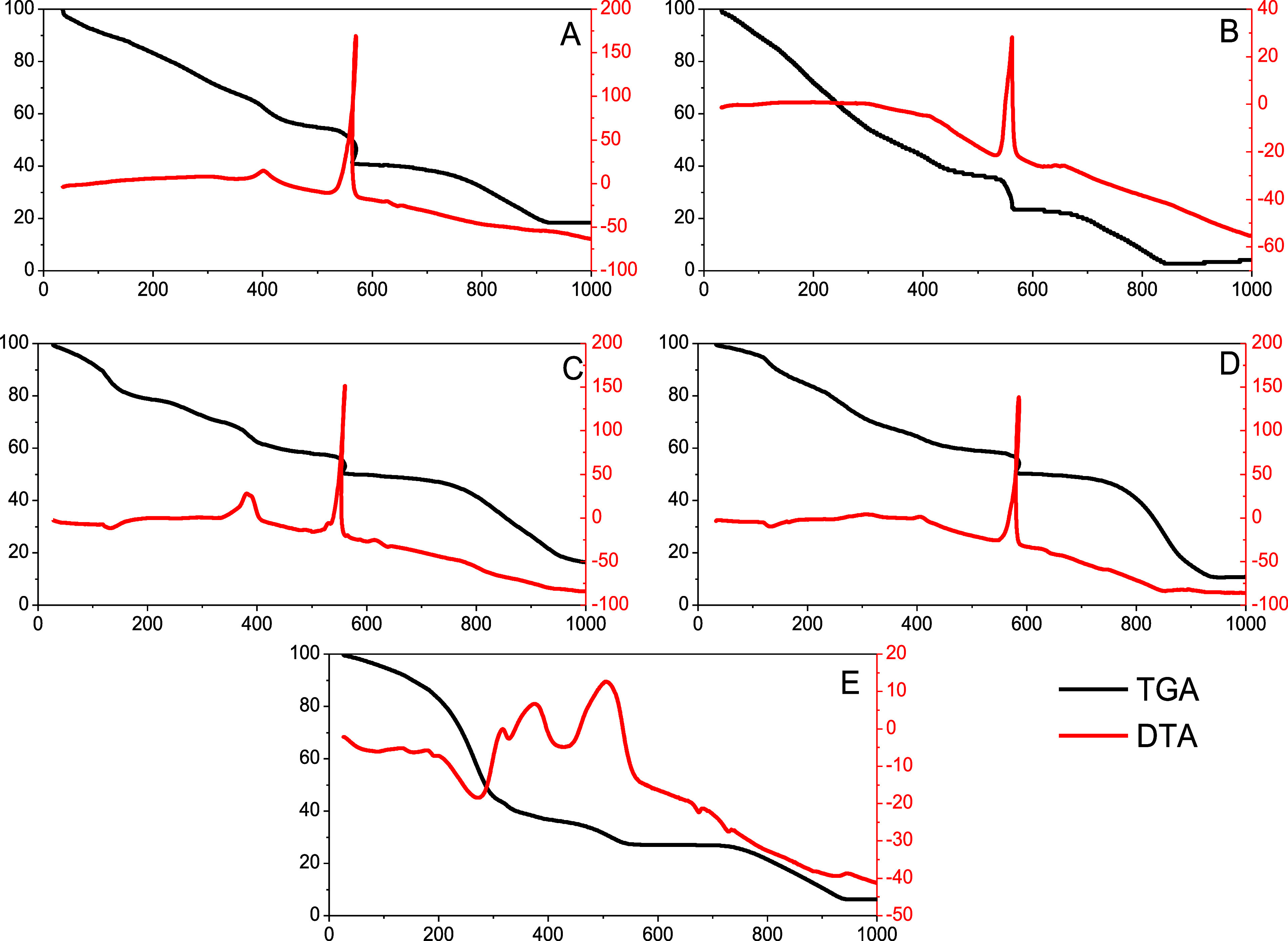
TG-DTA curves of vinasse (A), BCD-V 1 (B), BCD-V 2 (C), BCD-V 3 (D), and BCD-V 4 (E).

The BCD-V 3 samples notably displayed two distinct emission peaks when excited at 380 and 460 nm. This could be attributed to the presence of BioC-dots of varying sizes or possibly other organic substances within the vinasse matrix, highlighting the complexity and diversity of the organic compounds in this material. Time is an essential variable in nanocarbon formation, and it is observed that the QY increases with time. Additionally, the BCD-V 4 sample demonstrated the highest QY and significant peak displacement, reaching a maximum emission peak at 475 nm during excitation at 420 nm, as depicted in Figure 4. Alongside this, the calculated quantum yield (QY) for this synthesis markedly increased, achieving a value of 19.62%. This improvement in emission characteristics reflects the effective surface passivation of the synthesized BioC-dots. According to surface state theory, passivation can enhance emission by trapping excited electrons. Although the QY was calculated using excitation at 360 nm, we can infer by the functionalization results and the emission intensity that 24 h and functionalization presented. It shows that vinasse can produce high-intensity BioC-dots without adding doping materials to improve their emission intensity. Detailed emission parameters of the maximum registered emission for all samples are meticulously compiled in Table 2 for further reference, and their emission spectra are compared in Figure 4F.

**Table 2:** Emission and QY Data of the Vinasse and BCD-Vs

sample	λ_ex_/nm	λ_em_/nm	intensity	QY (%)
vinasse	460	530	7348	0.99
BCD-V 1	380	450	46,241,6	2.51
BCD-V 2	460	495	57,460	5.57
BCD-V 3	460	490	113,230	6.84
BCD-V 4	350	420	116,888,8	19.13

### 3.4 Thermal Analysis

The thermal degradation of the vinasse sample occurs in four distinct steps, as observed in Figure 4. The first step occurs between 36 and 337 °C, referring to the degradation of more volatile organic compounds in the sample and the dehydration of the vinasse, resulting in a weight loss of 31.1%. The relatively low degradation point of vinasse is attributed to the raw material’s nonfibrous composition, which lacks organic compounds in rigid structures, thereby reducing the overall energy required for degradation.^16^ The second degradation step occurs between 338 and 460 °C, attributed to the decomposition of more complex aliphatic organic materials, such as hemicellulose, carboxylic acids, and lignin, resulting in a 12.7% weight loss.^17^ The final thermal degradation step occurs between 460 and 925 °C, with a registered residue of 18.32%, indicating the presence of inorganic materials in the sample. The observed residue value corresponds with the already reported values in the literature.^18^

The TG-DTA curves of BCD-V 1 presented changes in the degradation curves, as observed in Figure 5. The decomposition of aliphatic compounds observed at 338–460 °C is no longer evident; instead, continuous sample degradation is now observed until 473 °C, with a more noticeable weight loss of 60.96%. The surface degradation of the BioC-dots also occurs at this temperature, as previously observed in other studies, with an increase in functional groups along the BioC-dot surface, providing a possible explanation for the change in the degradation profile. The sample also changed the degradation of the aromatic compound, observed between 520 and 590 °C, attributed to the coupled degradation with the BioC-dot core, with a registered weight loss of 12%. The last degradation step has a similar profile but presents lower registered residue values, at 3.42%, compared to 18.32% for the vinasse sample.

**Figure 5:**
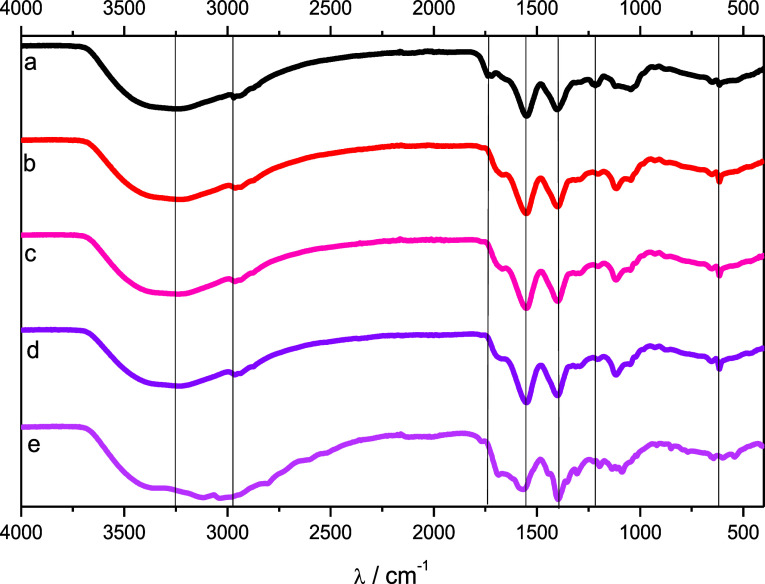
FTIR spectra of vinasse (A), BCD-V 1 (B), BCD-V 2 (C), BCD-V 3 (D), and BCD-V 4 (E).

The TG-DTA curves of BCD-V 2 presented small changes in the degradation curve, seen in the separation of the continuous degradation step between 36 and 337 °C in vinasse into two distinct steps, with water loss and volatile compounds occurring between 36 and 103 °C, with 9.3% weight loss, and the surface degradation of the BioC-dots being observed right after, identified by the endothermic point observed in 135 °C. The sample also presents alteration in the exothermic peak at 560 °C, possibly due to the interaction between the metals reported in the sample with the BioC-dots. The residue percentage at the end of the analysis was lower than that reported in vinasse (15.1%) but higher than the observed value in the 6 h synthesis.

The TG-DTA curves of the BCD-V 3 differ only in the last step of degradation, as the sample stabilizes at 943 °C instead of degrading until 995 °C. The inorganic residues found in the sample (10.67%) are even lower than the reported values in the BioC-dots synthesized for 12 h, indicating a better interaction between the present metals and the synthesized BioC-dots.

The TG-DTA curves of the synthesized BioC-dots presented small changes in their degradation, as observed in Figure 4. The first step until 360 °C shows two dehydrations of materials. Between 360 and 432 °C, the relative thermal degradation of nonconverted organic compounds to nanocarbon obtained during synthesis is proven by a significant mass loss at the product obtained in 6 h of synthesis. The third step between 532 and 600 °C in vinasse and BCD-V 2 and 3 can be considered the combustion, as observed by the typic exothermic peak in the DTA curve. This step is attributed to the external nanocarbon, corresponding to thermal degradation of the functional groups of nanomaterials. The last step occurs after 600 °C forwarded was altered by all synthesis times and is associated with the degradation of the BioC-dot nucleus.

### 3.5 FTIR Spectra

The vinasse FTIR spectra (Figure 5) presented notable absorption bands at 3250 cm^–1^, referring to the stretch of carboxylic and alcohol groups as the characteristic region of the hydrogen bonds in water molecules. The absorption band found at 2969 cm^–1^ refers to the stretch of C–H bonds of aliphatic compounds, while the observed band at 1720 cm^–1^ refers to the vibration of C = O bonds of carboxylic acids. At 1549 cm^–1^, the spectra are like those already recorded, as the literature data shows.^19^

The FTIR spectra of BCD-V 1, 2, 3, and 4 presented small changes that allowed for the confirmation of the presence of BioC-dots in the solution. It is observable in the spectra that a peak displacement from 1720 cm^–1^ to 1667 cm^–1^ indicates the degradation of carboxylic acids found in the vinasse. The appearance of a new absorption band at 1115 cm^–1^, referring to the vibration of C–O bonds in the BioC-dots surfaces, is also observed.

### 3.6 TEM Imaging

TEM imaging of BCD-V 3 revealed the formation of small clusters of various sizes, with a mean diameter of approximately 43.2 nm across the obtained solution, as shown in Figure 6A. Figure 6B shows the imaging of single BioC-dots with approximate sizes of 10–13 nm. The image of BCD-V 4 revealed the formation of clusters with diameters of approximately 50 nm, indicating a possible increase in the average BCD-V size.

**Figure 6:**
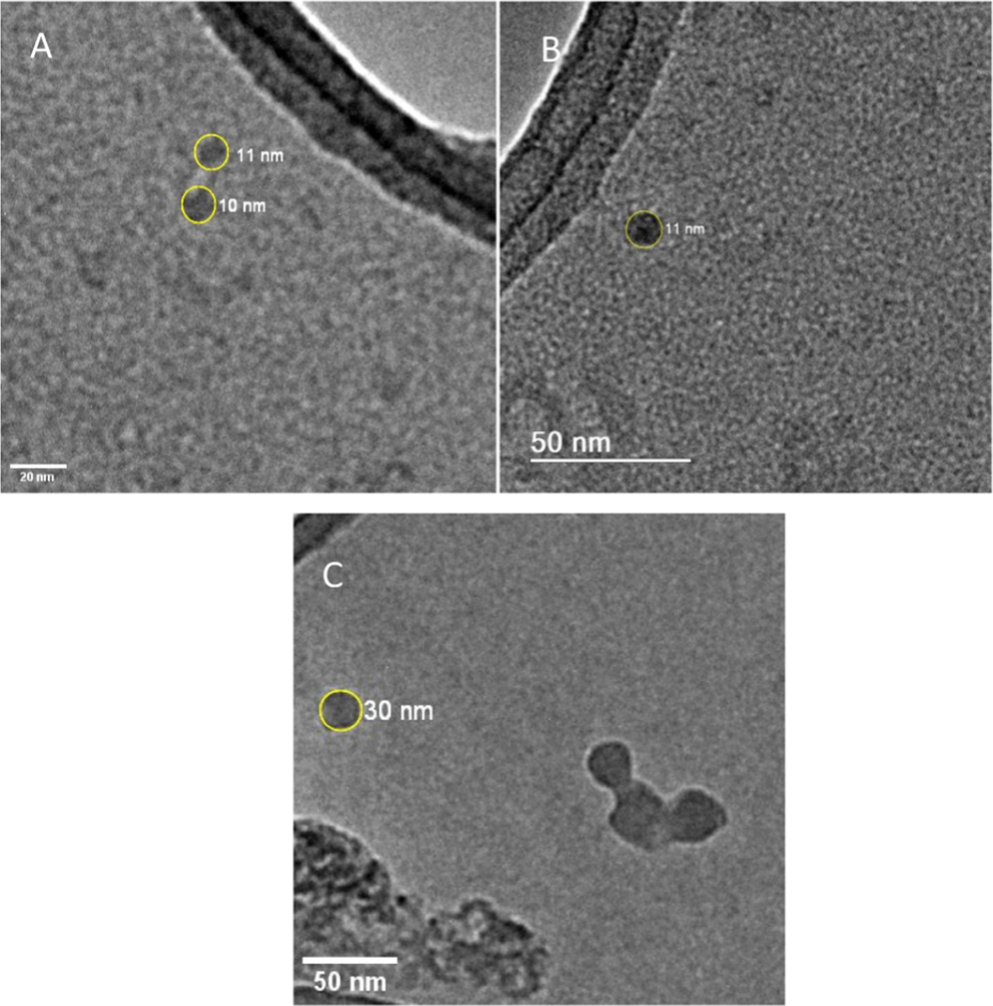
TEM image of BCD-V 3 (A,B) and BCD-V 4 (C).

### 3.7 Metal Detection

The addition of metal ions to BCD-V samples decreased the luminescence emission intensity, indicating that metal ion detection occurs via fluorescence resonance energy transfer (FRET). FRET occurs due to the transfer of energy from excited electrons in the carboxyl groups on the BCD-V surface to the metal ions, promoting the quenching of the emission system in solution with BCD-V.^20^ As shown in Figure 7, the Stern–Volmer relation between the added ions and BioC-dots solution was not linear, demonstrating that although there is a noticeable decrease, not every metal ion had access to the bonding sites in the BioC-dots surface. Analyzing the generated graph, we can observe that an increase in the concentration of cobalt­(II) and nickel­(II) ions in the BCD-V 4 sample produced a pronounced decrease in the sample intensity. In contrast, the increase of manganese­(II) ions did not change sample emission intensity significantly. The BioC-dots detected nickel­(II) ions with a calculated LOD of 18.22 mmol L^–1^, while cobalt­(II) ions were detected with a LOD of 13.57 mmol L^–1^. Analyzing the emission spectra of nickel­(II) and cobalt­(II) added solutions, the decrease in luminescence intensity occurs inversely logarithmically as the concentration of metal ions increases in the solution, as shown in Figure 8.

**Figure 7:**
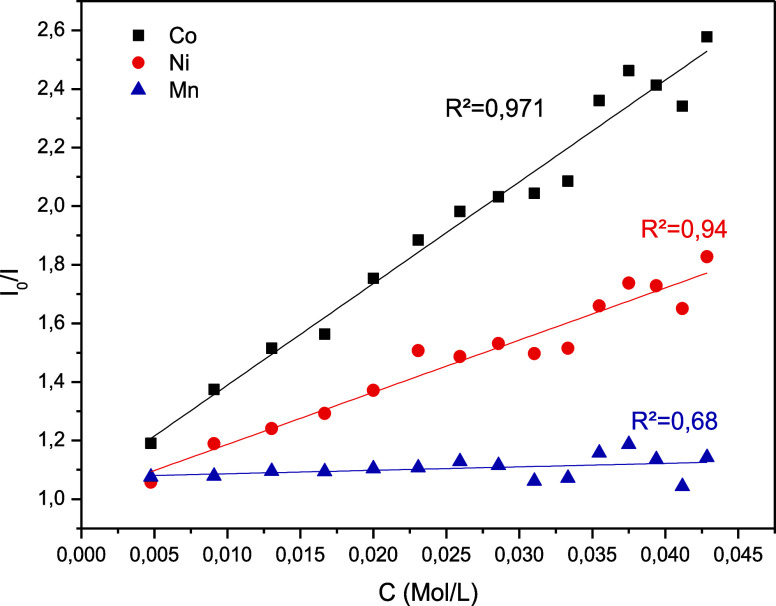
Effects of the presence of different metal ions in BCD-V solution.

**Figure 8:**
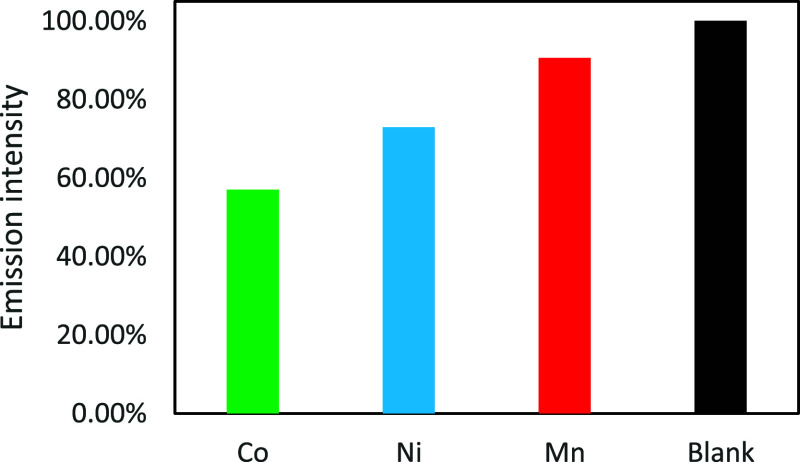
Effects of the presence of different metal ions in BCD-V solution.

As illustrated in Figure 8, there is a significant reduction in luminescence intensity when nickel­(II) and cobalt­(II) ions are exposed to UV light at a wavelength of 360 nm. The BioC-dots effectively detect nickel­(II) ions, with a limit of detection set at 18.22 mmol L^–1^. They also demonstrate the ability to detect Co­(II) ions at a lower limit of 13.57 mmol L^–1^. While the reported limit of detection (LOD) is lower than previous findings, it still presents significant advantages for potential portable sensing applications.^21^ The elevated LOD is particularly beneficial as it minimizes disruptions caused by contaminants, thereby enhancing reliability and precision in various environments.

A closer examination of the emission spectra for solutions containing both nickel­(II) and cobalt­(II) reveals that the decrease in luminescence intensity follows an inversely logarithmic relationship as the concentration of metal ions in the solution increases. This observation is further depicted in Figure 9, highlighting the correlation between the ion concentration and luminescence behavior. The implications of these findings suggest that as the concentration of these metal ions rises, there is a marked attenuation in the luminescent signal, which can be utilized for quantitative analysis of these ions in various applications.

**Figure 9:**
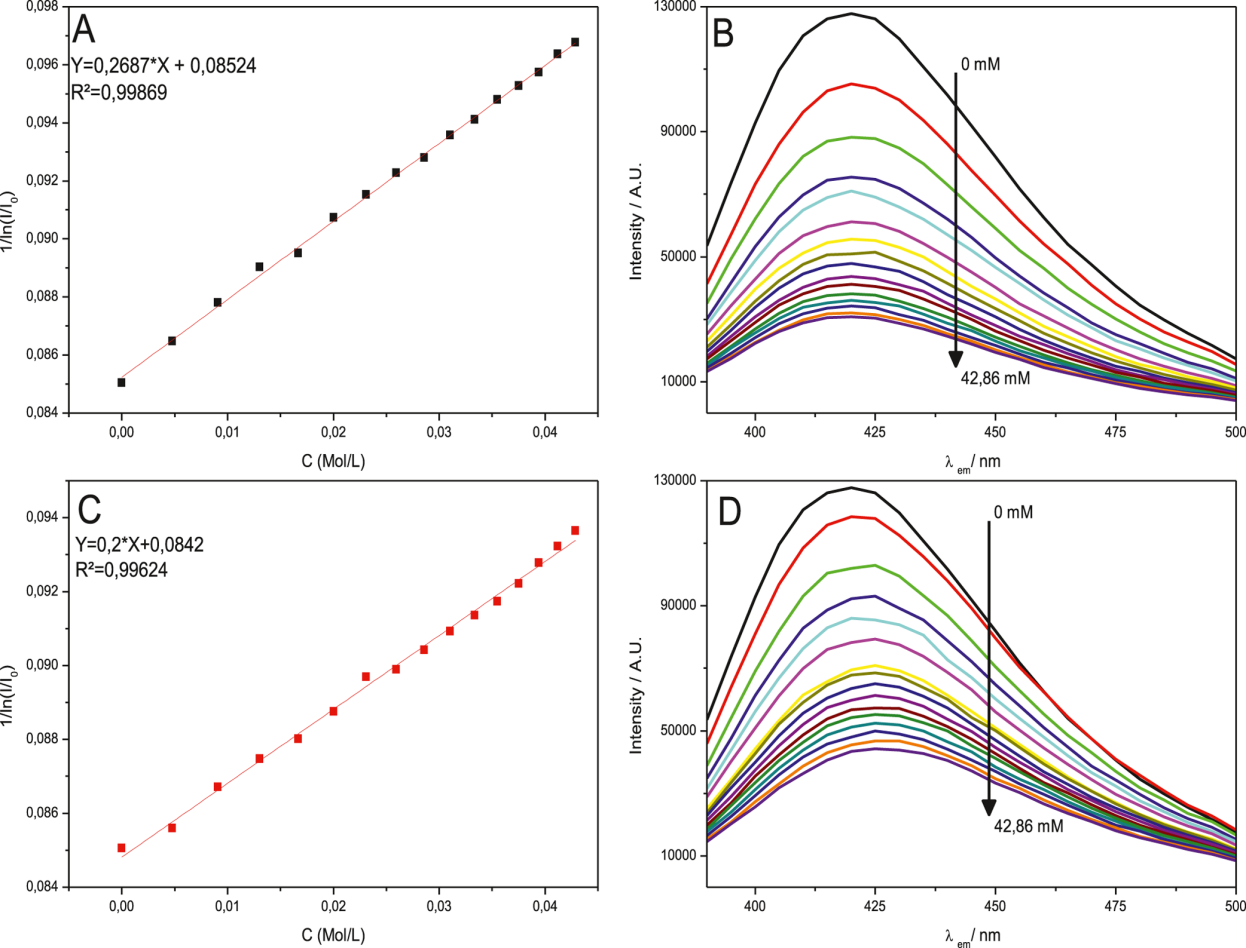
Relation between the maximum emission and ion concentration of cobalt­(II) (A) and nickel­(II) (C); decrease in emission intensity with the increase of metal ion concentration of cobalt­(II) (B) and nickel­(II) (D).

## 4 Conclusion

The ethanol distillation process yields a small quantity of luminescent nanocarbon. Although vinasse generates a modest amount of BioC-dots, the synthesis process effectively demonstrates wastewater refinement without pretreating the samples, resulting in nanomaterials with emissions within the 400–600 nm wavelength range, predominantly showcasing blue and green hues. The produced BioC-dots exhibit diameters of 10–13 nm. The physicochemical properties of these BioC-dots indicate that shorter synthesis times correlate with lower quantum yields, primarily due to incomplete material transformation. However, BCD-Vs’ functionalization leads to a notable increase in the quantum yield, attributed to the peak displacement caused by various functional groups on the BioC-dot surface. The BioC-dots produced showcase remarkable thermal stability and vibrant emission intensity, underscoring the extraordinary promise of vinasse in transforming wastewater into valuable biomaterials. Importantly, these BioC-dot samples possess the capability to detect Co­(II) and Ni­(II) ions, with impressive limits of detection at 13.57 and 18.22 mmol L^–1^, respectively, as demonstrated in the Stern–Volmer graph. This exemplifies the innovative potential of BioC-dots as a groundbreaking solution for the detection of metal ions.
